# Biosynthesized TiO₂ Nanoparticles to Enhance the Mechanical and Antibacterial Properties of Type-II Glass Ionomer Cement for Dental Restorative Applications

**DOI:** 10.7759/cureus.98281

**Published:** 2025-12-02

**Authors:** Shubha P, Harini KS, Ganesh S, Anubhav Jannu, Preethi Kusugal, Zarir Ruttonji

**Affiliations:** 1 Biomaterials Research and Development, Subbaiah Research Institute, Shimoga, IND; 2 Prosthodontics, Crown and Bridge, Subbaiah Institute of Dental Sciences, Shimoga, IND; 3 Prosthodontics, Crown and Bridge, Jagadguru Sri Shivarathreeshwara (JSS) Academy of Higher Education and Research, JSS Dental College and Hospital, Mysore, IND; 4 Oral and Maxillofacial Surgery, Subbaiah Institute of Dental Sciences, Shimoga, IND; 5 Prosthodontics, Crown and Bridge, Maratha Mandal's Nathajirao G. Halgekar Institute of Dental Sciences and Research Centre, Belagavi, IND

**Keywords:** antibacterial, compressive strength, glass ionomer, hydrothermal, l. acidophilus, microwave, setting time, s. mutans, titanium dioxide

## Abstract

Titanium dioxide (TiO₂) and its nanoforms are highly valued in dentistry for their biocompatibility, antimicrobial effects, and photocatalytic properties, making them useful functional additives in restorative materials. In this study, TiO₂ nanoparticles (TiO₂ NPs) were synthesized using bio-assisted methods and incorporated into type-II glass ionomer cement (GIC) to investigate their effect on the mechanical and antimicrobial performance of the cement. The modified GICs were evaluated for setting time, compressive strength, antimicrobial activity against *Streptococcus mutans* and *Lactobacillus acidophilus*, and antioxidant potential. The study explores whether incorporating bio-assisted TiO₂ NPs can enhance the functional and therapeutic properties of conventional GICs.

TiO₂ NPs were synthesized using *Piper betle* aqueous extract and *Ocimum sanctum* hydroalcoholic extract using hydrothermal and microwave-assisted synthesis methods, respectively. Synthesized TiO₂ NPs were subsequently subjected to physicochemical characterization and in vitro hemolysis and cytotoxicity assessments. Further, TiO₂ NPs were incorporated into type-II GIC powder to formulate four modified cement groups (F2-F5), along with an unmodified control group (F1), at two nanoparticle weight ratios. Compressive strength was measured using a universal testing machine (UTM) at a crosshead speed of 0.5 mm/min until the samples fractured. Antimicrobial activity was determined by exposing GIC discs (5 × 2 mm) to *S. mutans* (Microbial Type Culture Collection (MTCC) 890) and *L. acidophilus* (MTCC 10307) cultures, followed by plating and colony counting after incubation at 37°C for 24 and 72 hours. The antioxidant activity of F1-F5 formulations was also evaluated using the 2,2-diphenyl-1-picrylhydrazyl (DPPH) assay.

A significant reduction in the setting time was observed in GIC formulations containing 100 mg/g of TiO₂ NPs regardless of the synthesis method, with the difference in mean setting time between the control and TiO₂-incorporated groups being statistically significant (p < 0.05). Incorporation of 100 mg/g TiO₂ NPs also resulted in a notable increase in compressive strength (F3: 290 ± 11 MPa; F5: 301 ± 12 MPa), whereas the 50 mg/g formulations did not show statistically significant improvement. Antimicrobial evaluation revealed that even the control GIC exhibited low colony counts (<300) with no apparent increase after 72 hours; however, the addition of TiO₂ NPs provided a modest enhancement in antibacterial effects at both 24- and 72-hour timelines. TiO₂ NPs showed less than 20% antioxidant activity, likely due to residual phytoconstituents as indicated by Fourier transform infrared (FTIR) analysis. Formulations F3 and F5 displayed slightly higher antioxidant activity than the pure GIC (still <10%), although the differences were not considerable at the tested concentrations.

Overall, the results of our research indicate that plant-mediated synthesis resulted in TiO₂ NPs with favorable morphology, enhanced antibacterial effects, and good cytocompatibility. When incorporated into type-II GIC, TiO₂ NPs contributed to both increased compressive strength and decreased setting time. The TiO₂ NP-modified GICs also exhibited a slight improvement in antimicrobial activity and mild antioxidant effects, indicating their potential suitability as restorative materials after elaborate research on other required mechanical properties and pilot clinical performance.

## Introduction

Titanium dioxide (TiO₂) is a naturally occurring oxide of titanium that has significant biomedical applications due to its excellent biocompatibility, photocatalytic activity, and stability. It is extensively employed in dental implants, bone regeneration scaffolds, drug delivery systems, biosensing, and antimicrobial coatings [1]. Crystallographically, it exists in three distinct crystalline forms: anatase, rutile, and brookite. Among these, the anatase phase is highly preferred for biomedical applications due to its superior surface reactivity, ease of functionalization, photocatalytic efficiency, and antimicrobial properties [2,3].

TiO₂ and its nanoforms have found a wide range of applications in dentistry. It is used as a pigment in dentifrices, as an opacifying agent in dental composites, as a bioactive surface modifier on dental implant surfaces due to its osteoconductive nature, in tissue engineering, and as a regenerative material for osseous tissue regeneration, among others [4]. In recent years, TiO₂ nanoparticles (TiO₂ NPs) have also been explored for their potential applications as endodontic sealers, adhesives for orthodontic brackets, and fillers for composite resin restorative materials, as they are known to improve the mechanical properties, including strength, hardness, and wear resistance [5].

Among various restorative materials, particularly dental cements, glass ionomer cement (GIC) occupies a fundamental role in dentistry as it is a self-adhesive restorative material. Chemically, GIC combines fluoroaluminosilicate glass with polyacrylic acid, making it suitable for various restorative applications ranging from a luting agent to a direct restorative material in both conservative and pediatric dentistry [6]. Conventional GICs are widely used in routine dental restorative procedures due to their chemical adhesion to tooth structure, fluoride release, and biocompatibility; however, they present inherent limitations, which include low compressive strength, poor wear resistance, and high solubility in oral fluids. These drawbacks restrict their long-term performance, especially in stress-bearing posterior restorations [7].

Emerging evidence suggests that integrating nanomaterials with GIC can enhance both the fluoride-driven antibacterial effects and the mechanical resilience, thereby increasing their relevance in high-caries-risk clinical contexts [8]. In a recent study, TiO₂ NPs of size <25 nm were incorporated in a weight ratio of 3-5% in restorative GIC, significantly enhancing microhardness, strength, and antibacterial activity against* Streptococcus mutans *without affecting bond strength, indicating its potential as a durable antibacterial restorative material [9]. However, several studies have demonstrated that TiO₂ NPs could trigger oxidative stress, as a result of inflammation and DNA damage in aquatic species as well as mammals [10]. Conventional physical and chemical methods for NP synthesis often require high temperatures, toxic reagents, and costly equipment. Hence, recent research has shifted toward phyto-assisted synthesis, where plant extracts act as reducing and capping agents under mild, eco-friendly conditions, particularly beneficial for biomedical applications [11]. Since the TiO₂ NPs synthesized in this study are intended for long-term intraoral application, the authors selected two phytoextracts for the synthesis process. *Piper betle* aqueous extract and *Ocimum sanctum* hydroalcoholic extract were used for green synthesis, along with two energy sources, viz.,* *hydrothermal and microwave-assisted methods, respectively. These approaches were chosen to obtain highly crystalline anatase TiO₂ NPs. The resulting NPs demonstrated enhanced antimicrobial activity against *Streptococcus mutans* and *Lactobacillus acidophilus*, which are the primary organisms causing dental caries. Furthermore, TiO₂ NPs were intentionally synthesized with residual phytoconstituents from the plant extracts to explore their possible synergistic therapeutic effects as well as additional antioxidant benefits.

Accordingly, the objectives of this study were to (i) green-synthesize TiO₂ NPs using two different plant extracts and energy sources, (ii) characterize their physicochemical properties, and (iii) evaluate the setting time, compressive strength, antimicrobial activity, and antioxidant potential of type-II GIC modified with these TiO₂ NPs. To address these aims, the present investigation further examined whether incorporating green-synthesized TiO₂ NPs at a concentration of 100 mg/g (and less than that) into type-II GIC could enhance mechanical behavior, particularly compressive strength, while maintaining clinically acceptable setting characteristics, alongside improving antibacterial activity against two key cariogenic microorganisms. Based on this rationale, the authors hypothesize that the inclusion of green-synthesized TiO₂ NPs within type-II GIC will improve its compressive strength and antimicrobial efficacy without adversely affecting its setting profile. This structured approach is expected to provide a clear framework for evaluating the modified material's suitability for restorative dental applications.

## Materials and methods

The synthesis, physicochemical characterization, and toxicity assessment of TiO₂ NPs synthesized via hydrothermal and microwave-assisted methods are detailed in the Appendices section. The primary manuscript includes experimental methodologies, findings, and a discussion on the integration of these biosynthesized TiO₂ NPs into type-II GIC powder.

Effect of the incorporation of biosynthesized TiO₂ NPs on the mechanical properties of type-II GIC

For assessing the effect of incorporating biosynthesized TiO₂ NPs on mechanical and antibacterial properties, type-II GIC powder (GC Fuji II, Universal Restorative, Japan, batch number: 2305021) was used. Even though the exact composition of the powder and liquid used in the experiments, along with the concentrations, is not available, the powder part of type-II GIC contains fluoroaluminosilicate glass, polyacrylic acid, and pigments, and the liquid contains polyacrylic acid, distilled water, and polybasic carboxylic acid. The TiO₂ NPs synthesized using hydrothermal and microwave-assisted methods (as described in the Appendices section) were added to the powder of GIC at two different concentrations. The details of the formulations are given in Table 1.

**Table 1:** Type-II GIC formulations containing various ratios of biosynthesized TiO2 NPs F1: control; F2: 50 mg/g of hydrothermal TiO₂ NPs mixed to GIC; F3: 100 mg/g HT TiO₂ NPs mixed to GIC; F4: 50 mg/g of MV TiO₂ NPs mixed to GIC; F5: 100 mg/g of MV TiO₂ NPs mixed to GIC Statistical significance is set at p < 0.05 Quantity of NPs and GIC in grams GIC: glass ionomer cement; HT: hydrothermal; MV: microwave; TiO₂: titanium dioxide; NPs: nanoparticles

Ingredient name	F1	F2	F3	F4	F5
Fuji II-GIC powder	1	1	1	1	1
TiO₂(HT synthesized)	-	0.05	0.1	-	-
TiO₂(MV synthesized)	-	-	-	0.05	0.1

A total of four cement formulations (F2-F5) were prepared for further testing. The F1 formulation served as a control, consisting of GIC powder without the incorporation of NPs.

To prepare the test batch formulations F2-F5, 1 g each of GIC powder was dispensed into a clean and dry dappen dish. A predetermined quantity of TiO₂ NPs was carefully measured (as specified in the batch/group mentioned in Table 1) and added to the GIC powder in the dappen dish. Both powders (GIC and TiO₂ NPs) were manually mixed using a dry agate spatula for 2-3 minutes in both clockwise and anticlockwise directions to ensure proper mixing. This physical mixture was further used as the "powder" part of the cement in the future experiments.

Determination of Setting Time

GIC is a fast-setting cement and typically sets within 2-3 minutes after mixing the powder with the liquid [6]. To assess how TiO₂ NPs affect the setting time, 1 g of each formulation (F1-F5) was mixed with the manufacturer-provided GIC liquid using the folding technique. Each formulation was mixed to a restorative consistency. The mixtures were then evaluated for mixing time, setting time, and the formation of a plastic mass suitable for condensation into the prepared cavity.

The setting time was measured following the protocol of Hemmati et al., with major modifications because the equipment specified in the original method was not available during this study [12]. The sample size was determined using one-way analysis of variance (ANOVA) for five independent groups (F1-F5), with a significance level of p < 0.05 and a desired power of 0.80. Since no prior data existed on the variability of setting time in TiO₂-modified GIC, standard ANOVA effect-size benchmarks (Cohen's f) were used for sample planning.

**Figure 1:**
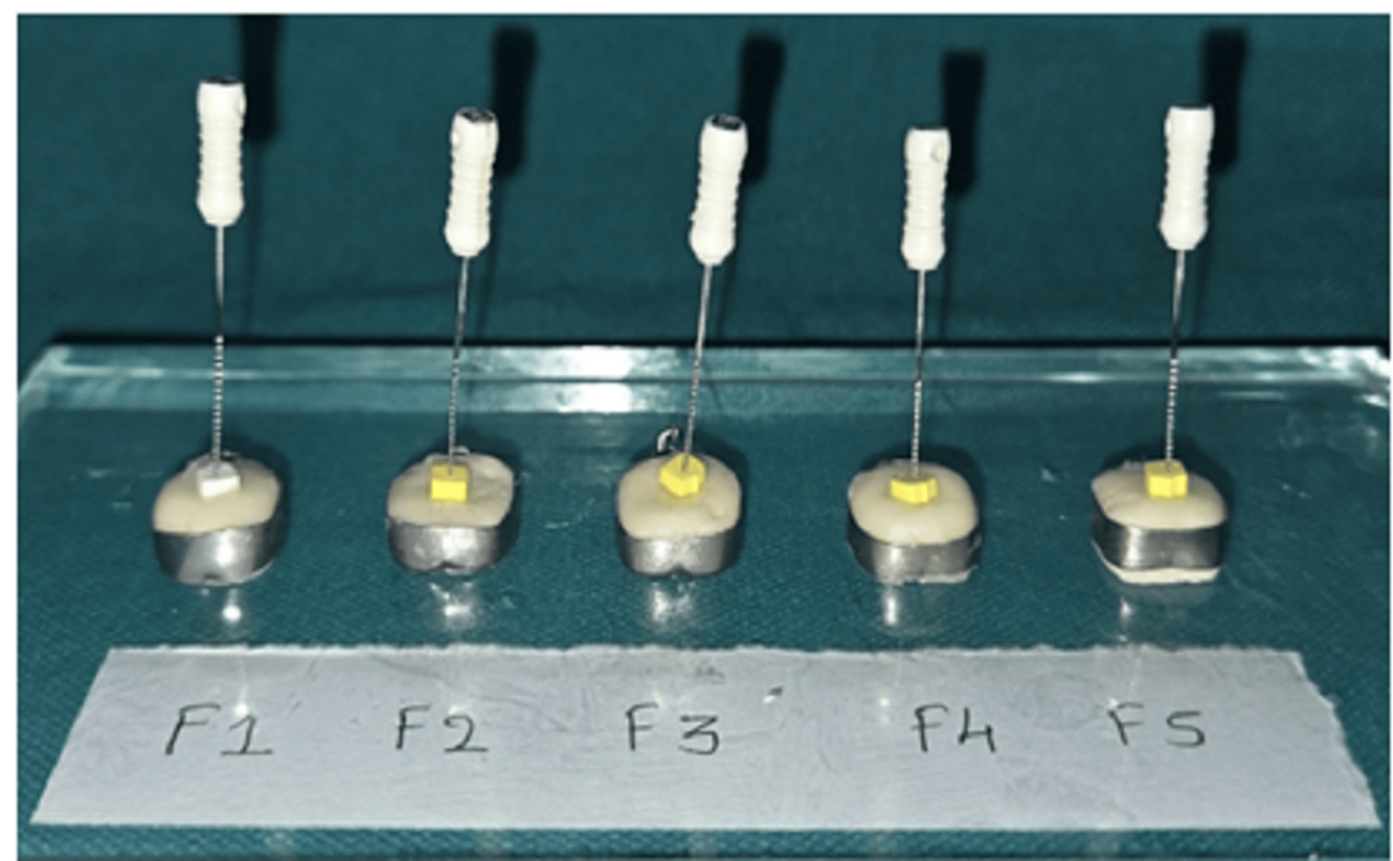
Determination of the setting time of TiO2 NPs incorporated into glass ionomer cement using the modified endodontic K-file NPs: nanoparticles; TiO₂: titanium dioxide

Briefly, 25 numbers of stainless steel (SS) orthodontic bands (n = 25) of diameter 5 mm were taken. F1-F5 cement formulations were mixed with polyacrylic acid liquid provided by the manufacturer to restorative consistency on a mixing pad and loaded to SS orthodontic bands using a dry spatula as shown in Figure 1. 

In the original experiment by Hemmati et al., an ISO 6876:2001-based rheometric indenter was used to measure penetration depth at 30-second intervals [12]. In the present study, this instrument was not available. Therefore, we modified a size 50 endodontic K-file to perform the setting time experiment.

The K-file tip was shortened by 1 mm using a tungsten carbide bur to create a flat, blunt surface similar to the ISO-recommended indenter. The modified instrument was then gently pressed into different areas of the cement specimen every 30 seconds. A standardized one-finger pressure (approximately 5 ounces) was applied. Indentations were made until the instrument no longer penetrated more than 3.5 mm. The time point just before this occurred was recorded as the setting time.

This modified method preserves the purpose of the original protocol: monitoring the reduction in penetrability as the cement hardens while offering a reproducible alternative with the equipment available.

Determination of Compressive Strength

**Figure 2:**
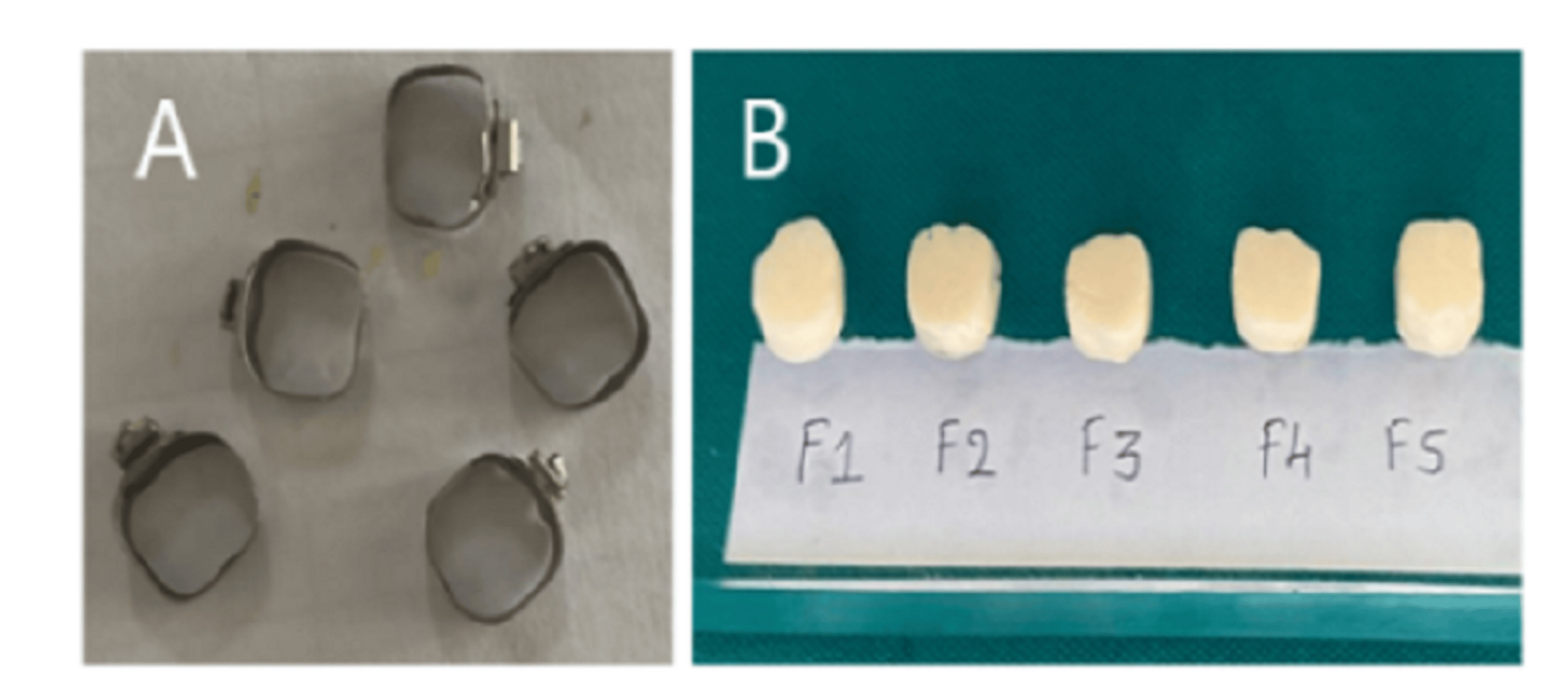
Stainless steel orthodontic bands are used to prepare specimen for compressive strength testing (A). Test specimens of TiO₂ incorporated into GIC were prepared using orthodontic bands (B) GIC: glass ionomer cement; TiO₂: titanium dioxide

For preparing the specimens to test the compressive strength of F1-F5 formulations, an SS orthodontic band of size 7.5 mm × 2 mm was used. F1-F5 cement formulations were mixed to restorative consistency and loaded into these bands. A total of 25 specimens were prepared for compressive strength measurement (statistically justified as above, in the setting time experiments). Figure 2 shows the cement specimens' preparation for compressive strength testing.

After 24 hours of filling the specimen into the mould, the specimens were gently removed from the pattern and kept immersed in distilled water for two hours. Further, the specimens were kept in a hot air oven at 37°C for one day. The compressive strength of the test specimens was determined using a universal testing machine (UTM) with a claw of 2 cm diameter. The crosshead speed was fixed to 0.5 mm/min until the specimen fractured.

The compressive strength was calculated using the formula $\text{CS}=\frac{4\text{F}}{\pi{}d^{2}}$ where F is the maximum load applied and d is the diameter of the specimen. The obtained data were statistically analyzed using one-way ANOVA at a significance level of p < 0.05 [13]. The values were converted to MPa by multiplying each result by the conversion factor 9.81.

Determination of the biological activity of TiO₂ NPs incorporated into type-II GIC

*Determination of the Antimicrobial Activity of TiO*₂* NPs Incorporated Into GIC Against Cariogenic Microorganisms*

Ten circular cement specimens (n = 10; total 50 specimens) of 5 × 2 mm (F1-F5 formulation) dimensions were prepared using an SS orthodontic band. After dipping the cement specimens in distilled water for 24 hours, the specimens were gently dried with a paper towel and kept at 70°C in a hot air oven for complete drying for two hours. Further, the specimens were packed inside lidded bottles filled with artificial saliva and ethylene oxide (EtO) sterilized under controlled humidity, 37-63°C chamber temperature, and vacuum-assisted EtO gas exposure, followed by aeration for one hour and an evacuation of 10 cycles to remove excess EtO gas. Further, these sterile specimens were kept at room temperature for 24 hours.

The *S. mutans* (Microbial Type Culture Collection (MTCC) 890) inoculum was prepared in brain heart infusion (BHI) broth, and the *L. acidophilus* (MTCC 10307) inoculum was prepared in de Man, Rogosa and Sharpe (MRS) broth. The turbidity of both cultures was adjusted to 1 × 10⁶ CFU/ml using a 0.5 McFarland standard.

From each inoculum, 1 ml was transferred into a sterile 2 ml Eppendorf tube. Inside a laminar airflow hood, 1 ml of artificial saliva, presumed to contain TiO₂ NPs leached from the F2 to F5 cement formulations, was added. The tubes were incubated at 37°C for 24 hours. No serial dilution was performed.

After incubation, the tubes were checked visually for turbidity changes. Then, 20 µl from each tube was plated on BHI agar for *S. mutans* and on MRS agar for *L. acidophilus*. The plates were incubated at 37°C for 24 hours. All experiments were conducted in triplicate, giving a total of 15 plates for the F1-F5 groups.

A second set of plates received the same inoculum volume and was incubated for 72 hours. According to the American Society for Testing and Materials (ASTM) guidelines, the acceptable colony range for spread plate assays is 25-250 colonies [14]. After incubation, colonies were counted using a digital colony counter. Mean values and standard deviations were calculated and reported using IBM SPSS Statistics for Windows, V. 30.0 (IBM Corp., Armonk, NY, USA).

*Determination of the Antioxidant Activity of TiO*₂* NPs Incorporated Into Type-II GIC Formulations *

One set of circular cement specimens from the F1 to F5 groups, each weighing about 4.5 g, was used for the antioxidant assay. These specimens were ground into a fine powder using an agate mortar. Approximately 0.5 g of the powder was placed into a 2 ml Eppendorf tube. Then, 2 ml of sterile artificial saliva was added, and the mixture was probe-sonicated for 30 minutes.

In five separate Eppendorf tubes, 0.5 ml of a freshly prepared 0.1 mM 2,2-diphenyl-1-picrylhydrazyl (DPPH) solution was taken. DPPH is a stable free radical with an intense purple color and a maximum absorption at 517 nm [15]. To each tube, 0.5 ml of the cement dispersion (F1-F5) was added. The contents were vortexed for one minute to ensure proper mixing.

The reaction mixtures were incubated inside an opaque box for 20 minutes with continuous shaking at room temperature. After incubation, absorbance was measured at 517 nm using an ultraviolet-visible (UV-Vis) spectrophotometer (ELICO-SA 165, Elico Ltd., Hyderabad, India). The percentage of radical scavenging was then calculated using the standard formula $\%\,\text{DPPH scavenging activity} = \frac{A_c - A_s}{A_c} \times 100$ where Ac is the absorbance of the control and As is the absorbance of the sample. The 0.2% ascorbic acid solution was used as the standard antioxidant for comparison.

## Results

Effect of the incorporation of biosynthesized TiO₂ NPs on the mechanical properties of type-II GIC

Determination of Setting Time

**Figure 3:**
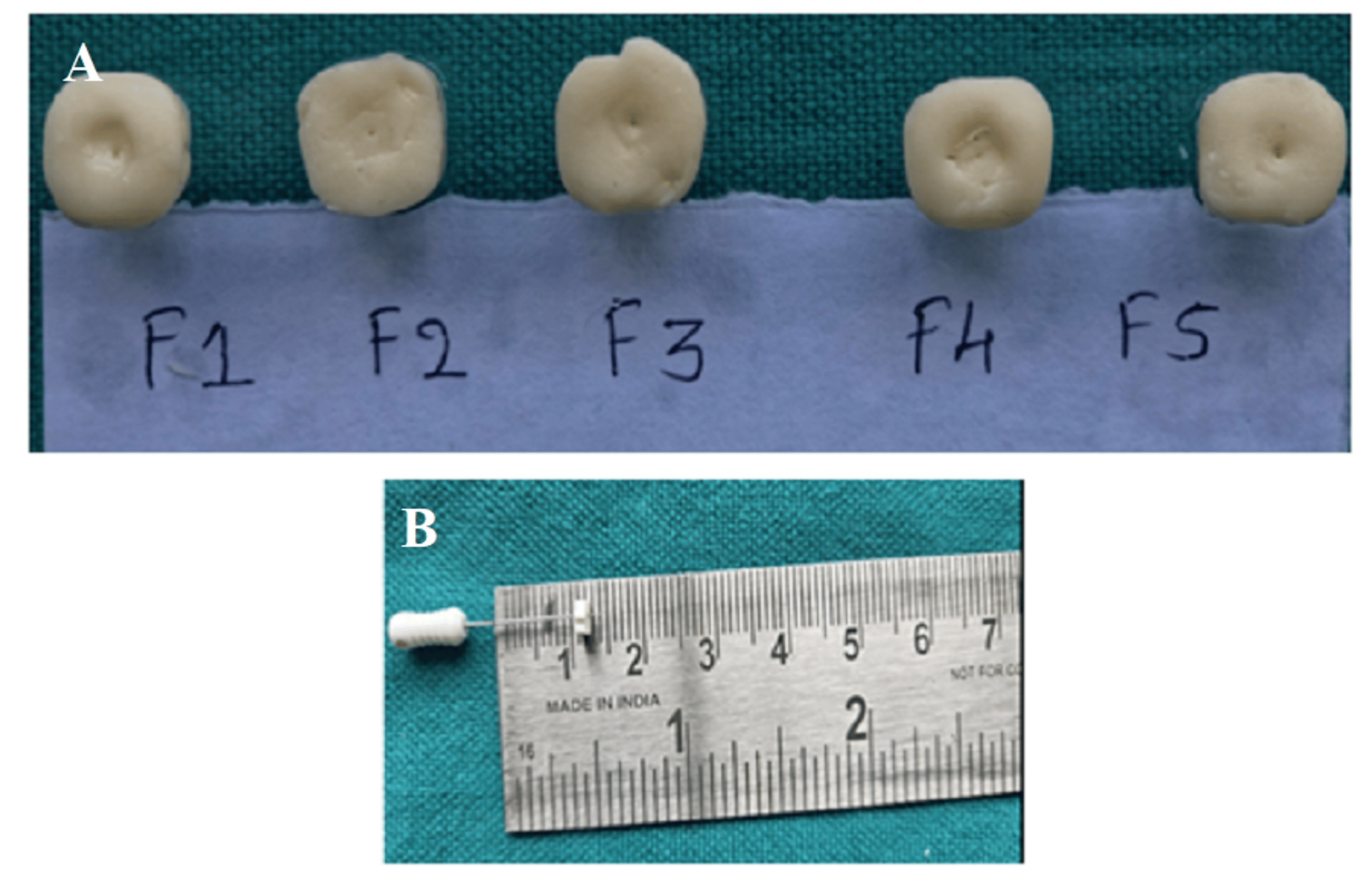
Indentations made by the penetrating file inside the cement specimen from one to three minutes (A). Measurement of the file's penetration depth using a stopper attached to the K-file (B)

Type-II GIC is a quick-setting cement with a setting time of 2-3 minutes. In the present work, an initial setting time determination experiment was carried out for a duration of 10 minutes (until the 50 size endodontic K-file could no longer penetrate more than 3.5 mm within the cement matrix). Figure 3 shows the depth of penetration by the endodontic file inside F1-F5 formulations.

Table 2 shows the setting time demonstrated by GIC incorporated with TiO₂ NPs synthesized by bio-assisted methods. From the table, it was found that there was a significant decrease in the setting time of GIC formulations incorporating 100 mg/g of TiO₂ NPs, irrespective of the synthesis method and particle size (hydrothermal or microwave-assisted synthesis). Independent t-test analysis (p < 0.05) showed that GIC formulations containing 100 mg/g of TiO₂ NPs (F3 and F5) had a significantly lower setting time compared with the control (F1). Groups with lower nanoparticle concentrations (F2 and F4) did not differ significantly from the control. Thus, the reduction in setting time is attributable to the higher TiO₂ NP loading, regardless of the synthesis method.

**Table 2:** Setting time of GIC incorporated with TiO2 NPs in different concentrations Statistical significance is set at p < 0.05 GIC: glass ionomer cement; NPs: nanoparticles; TiO₂: titanium dioxide

Group name	Setting time (in min)
F1	2.7 ± 0.52
F2	2.4 ± 0.56
F3	1.9 ± 0.61
F4	2.7 ± 0.63
F5	1.7 ± 0.59

Determination of Compressive Strength

The compressive strength of F1-F5 cement formulations was carried out in triplicate, and the average compressive strength was reported in megapascals (MPa). Table 3 shows the results of the compressive strength of cement specimens incorporated with TiO₂ NPs after 24 hours of setting. Independent t-test analysis (p < 0.05) showed that only the groups containing 100 mg/g of TiO₂ NPs (F3 and F5) exhibited a statistically significant increase in compressive strength compared with the control (F1). The groups with 50 mg/g TiO₂ NPs (F2 and F4) did not show any significant difference from the control (p > 0.05). Thus, the enhancement in compressive strength is attributable solely to the higher nanoparticle loading, irrespective of the synthesis method.

**Table 3:** Compressive strength of F1-F5 cement formulations Statistical significance is set at p < 0.05

Group name	Compressive strength (MPa)
F1	252 ± 14
F2	254 ± 19
F3	290 ±11
F4	254 ± 17
F5	301 ± 12

From the previous literature, it has been observed that the Fuji type-II GIC has a compressive strength of around 252 MPa after 24 hours of mixing [16]. Results obtained from our studies show that there was a significant improvement in the compressive strength of the cement specimen following the incorporation of 100 mg/g of TiO₂ NPs, irrespective of their synthesis method. The 50 mg/g TiO₂ NPs did not demonstrate any statistically significant result.

Determination of the biological activity of TiO₂ NPs incorporated into type-II GIC

*Antimicrobial Activity of TiO*₂* NPs Incorporated Into Type-II GIC Against Dental Caries-Causing Organisms*

The antibacterial activity of F1-F5 cement formulations was determined against *S. mutans *and *L. acidophilus*, the major pathogens causing dental caries. The antibacterial activity tests were performed after 24 hours and 72 hours of incubation to determine the possible release of TiO₂ NPs into the artificial saliva, which may cause the sustained effect. F1 formulation without the incorporation of NPs was used as a control. Table 4 and Table 5 show the antibacterial activity of TiO₂ NPs against *S. mutans* after 24 hours of incubation.

**Table 4:** Antibacterial activity of F1-F5 formulations against S. mutans after 24 hours of initial setting reaction Statistical significance is set at p < 0.05

Average number of colonies	F1	F2	F3	F4	F5
Trial I	112	100	100	102	90
Trial II	110	100	99	97	89
Trial III	110	98	100	101	89
Mean	111.33	99.3	99.6	100.00	90.33
Standard deviation	1.15 ± 0.08	1.15 ± 0.06	0.57 ± 0.02	0.57 ± 0.04	0.57 ± 0.07

**Table 5:** Antibacterial activity of F1-F5 formulations against L. acidophilus after 24 hours of initial setting reaction Statistical significance is set at p < 0.05

Average number of colonies	F1	F2	F3	F4	F5
Trial I	112	108	106	107	95
Trial II	110	112	95	110	89
Trial III	110	107	105	107	91
Mean	111.33	109.33	102.00	108.00	91.66
Standard deviation	1.15 ± 0.08	1.17 ± 0.06	0.46 ± 0.02	0.50 ± 0.04	0.52 ± 0.07

Table 6 and Table 7 show the antimicrobial activity of the GIC + TiO₂ formulation after 72 hours of setting. It can be noted that the results remained consistent with a marginal increase in antimicrobial activity post-incorporation of TiO₂ NPs.

**Table 6:** Antibacterial activity of F1-F5 formulations against S. mutans after 72 hours of initial setting reaction Statistical significance is set at p < 0.05

Average number of colonies	F1	F2	F3	F4	F5
Trial I	117	114	96	112	90
Trial II	117	113	94	105	91
Trial III	118	113	94	110	91
Mean	111.33	113.33	99.6	109.00	90.33
Standard deviation	1.15 ± 0.08	1.15 ± 0.06	0.57 ± 0.02	0.57 ± 0.06	0.57 ± 0.04

**Table 7:** Antibacterial activity of F1-F5 formulations against L. acidophilus after 72 hours of initial setting reaction Statistical significance is set at p < 0.05

Average number of colonies	F1	F2	F3	F4	F5
Trial I	117	111	97	112	91
Trial II	117	110	92	105	86
Trial III	118	109	92	110	86
Mean	111.33	110.00	93.6	109.00	87.66
Standard deviation	1.15 ± 0.08	1.17 ± 0.06	0.49 ± 0.02	0.46 ± 0.06	0.57 ± 0.04

Among dental cements, GIC is known to have antimicrobial activity, fluoride release, chemical adhesion to tooth structure, and biocompatibility [17].

The results showed that even without TiO₂ NPs, all formulations had colony counts well below the 300-colony limit. After 72 hours, no increase in colony count was observed. The addition of TiO₂ produced only a marginal improvement in antibacterial activity.

Statistical analysis indicated a small but significant reduction in colony counts for both *S. mutans* and *L. acidophilus* at 24 hours and 72 hours when 100 mg/g TiO₂ was incorporated. F5 showed the lowest colony counts and a significant difference from the control (F1). F3 also showed a significant reduction, though less than F5.

The 50 mg/g groups (F2 and F4) did not differ significantly from the control (p > 0.05). Overall, TiO₂ provided only a slight additional antibacterial effect in the considered experimental range.

*Determination of the Antioxidant Activity of TiO*₂* NPs Incorporated Into Type-II GIC*

The antioxidant activity of F1-F5 formulations was determined using the DPPH free radical scavenging assay. DPPH free radical scavenging activity is a measure of the antioxidant ability of materials. DPPH free radicals produce a characteristic absorption at 517 nm. In the current work, it was noted that the GIC had a very negligible antioxidant activity.

**Figure 4:**
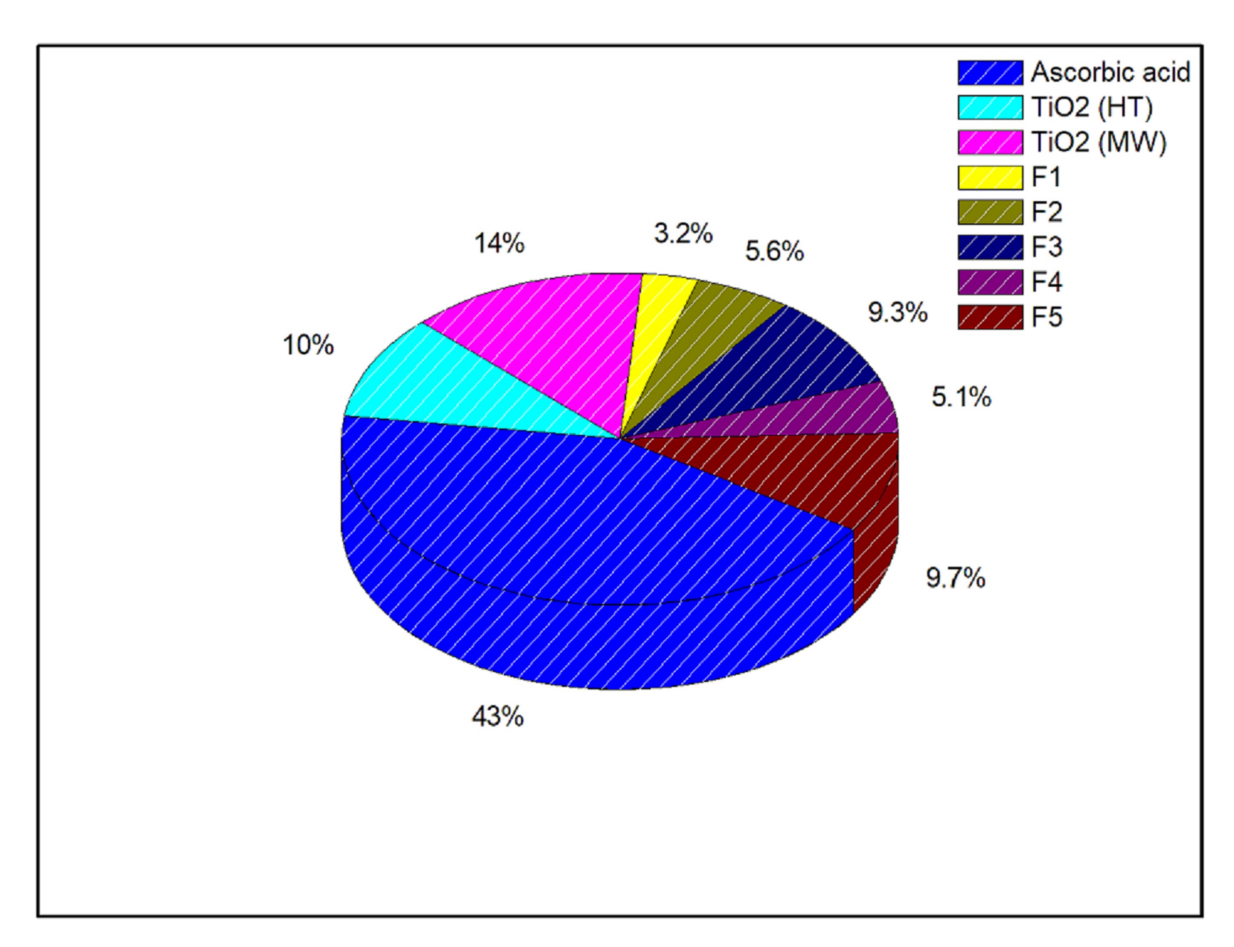
Percentage of DPPH free radical scavenging by various glass ionomer cement formulations and biosynthesized TiO2 NPs DPPH: 2,2-diphenyl-1-picrylhydrazyl; NPs: nanoparticles; TiO₂: titanium dioxide; HT: hydrothermal; MV: microwave

Biosynthesized TiO₂ NPs showed less than 20% antioxidant activity, probably due to the presence of remnants of phytoextracts used in their synthesis, as confirmed by the Fourier transform infrared spectroscopy (FTIR) results (see Appendices). The F3 and F5 formulations showed little more antioxidant activity than pure cement (less than 10%); however, the results are not very significant at these concentrations (Figure 4).

## Discussion

In the current research, TiO₂ NPs were synthesized using both hydrothermal and microwave-assisted methods, employing *P. betle* aqueous extract and *O. sanctum* hydroalcoholic extract, respectively. The influence of synthesis route, particle size, and morphology on the biological activity of TiO₂ NPs was evaluated, and the synthesized NPs were incorporated into type-II GIC to assess their effect on setting time, compressive strength, and antimicrobial activity against cariogenic microorganisms; furthermore, preliminary antioxidant activity was determined. The findings demonstrate that bio-assisted synthesis can yield biocompatible and functional TiO₂ NPs capable of enhancing the mechanical and biological performance of dental restorative materials.

When incorporated into type-II GIC, the biosynthesized TiO₂ NPs produced notable improvements in key functional properties. At higher loading (100 mg/g), TiO₂ served as nucleation centers that compacted the cement matrix, reduced porosity, and enhanced structural cross-linking. This resulted in a significant reduction in setting time (F3 and F5: 1.9 ± 0.61 and 1.7 ± 0.59 min vs. 2.7 ± 0.52 min for the control) and a marked increase in compressive strength (F3 and F5: 290 ± 11 and 301 ± 12 MPa vs. 252 ± 14 MPa for F1). In contrast, formulations containing 50 mg/g TiO₂ (F2 and F4) showed no statistically meaningful changes, indicating that the reinforcement effect becomes prominent only at higher nanoparticle concentrations. These findings are consistent with earlier reports demonstrating that TiO₂ NP additions enhance microhardness and mechanical reinforcement by creating additional nucleation sites and refining microstructure.

When the synthesis method is analyzed (see Appendices), the bio-assisted method has significantly influenced the physicochemical properties and biological activities of TiO₂ NPs. Upon advanced characterization of biosynthesized TiO₂ NPs, the X-ray diffraction (XRD) studies demonstrated broader and less intense XRD peaks, indicating a smaller crystallite size and the presence of surface-bound organic groups. Such surface functionalization induces internal lattice strain, lowers peak intensity, and enhances colloidal stability [17]. Microwave-assisted synthesis process further accelerated nucleation and crystal growth, leading to the formation of short rod-like anatase TiO₂ NPs. The hydroalcoholic extract of *O. sanctum*, rich in metal-chelating polyphenolics, likely directed anisotropic growth by selectively binding to specific crystallographic faces, thereby controlling nanoparticle shape [18].

The influence of particle size and surface chemistry has significantly affected the biological activity of TiO₂ NPs. Smaller particles possess higher surface reactivity and generate more reactive oxygen species (ROS) under light exposure [19]. In the present work, trace amounts of phytoorganics were intentionally retained on the nanoparticle surface to facilitate visible-light-based antibacterial activity [20]. The flavonoids present in the *P. betle* aqueous extract and the *O. sanctum* hydroalcoholic extract exhibit membrane-stabilizing properties [21]. This likely contributed to the lower hemolytic response observed in the biosynthesized TiO₂ NPs compared to commercially sourced TiO₂ NPs (see Appendices), indicating improved antibacterial efficiency and better biocompatibility. From the scanning electron microscopy (SEM) results, it was evident that *P. betle* extract promoted the partial coalescence of NPs, consistent with previous research work [22], and *O. sanctum* hydroalcoholic extract promoted the formation of well-defined short nanorods, an anisotropic morphology known to alter biological interactions compared to spherical particles [23]. Agglomeration patterns were mainly affected by the pH of the plant extracts used in the synthesis process.

TiO₂ NPs' incorporation into type-II GIC positively influenced both the mechanical and biological activity of the base cement. Due to their extremely small size, TiO₂ NPs might serve as nucleation centers, facilitating a compact cement matrix and minimizing porosity formation, which has resulted in improved compressive strength without much affecting the setting time, as seen from the results of setting time and compressive strength [24].

Although the present study did not evaluate fluoride release kinetics, earlier investigations have shown that TiO₂ addition may reduce pore size within the cement structure, which could help stabilize fluoride release patterns and potentially prolong cariostatic effects [8]. This contextualizes our mechanical findings within the broader understanding of how nanoparticle-mediated microstructural refinement can support long-term material performance.

Similar strengthening effects have been reported in the literature, where TiO₂-modified GICs showed increased microhardness and structural integrity, attributed to enhanced diffusion-controlled cross-linking and the availability of additional nucleation sites [25,26]. Compared with previous studies that used higher nanoparticle loadings or different filler chemistries, our results indicate that controlled incorporation of biosynthesized TiO₂ NPs provides mechanical reinforcement while maintaining adequate workability of the GIC matrix [27].

The TiO₂ NPs synthesized by both hydrothermal and microwave-assisted methods exhibited antibacterial activity primarily through the generation of ROS, disrupting the bacterial cell membranes and components [28].The TiO₂ NPs (rods) synthesized by the microwave-assisted method showed greater surface reactivity due to intentionally left phytoorganics, likely showing enhanced visible-light-assisted antibacterial effects [29]. 

The TiO₂ NPs synthesized by the bio-assisted method showed a very minimal antioxidant activity against DPPH free radical, probably due to the presence of residual flavonoids and polyphenols from the plant extracts, which can donate electrons to neutralize free radicals. This would be another additional advantage due to capping from biomolecules of phytoextracts that result in improved antimicrobial action along with mild antioxidant activity [23]_. _In line with Ramic et al., who demonstrated that TiO₂ NP-modified GICs maintain biocompatibility while exhibiting enhanced antibacterial performance, our study further confirms that TiO₂ NPs contribute positively to the biological activity of the base cement through ROS-mediated antimicrobial effects [30]. 

Limitations and the future scope of the work

Even though the TiO₂ NPs synthesized by bio-assisted methods showed promising antibacterial activity against two dental caries-causing organisms, along with biocompatible properties, the precise mechanisms of shape formation and surface functionalization were not fully clarified. Additionally, in the microbiology work, antimicrobial assessment was based on a single inoculum concentration and did not include serial dilution-based minimum inhibitory concentration (MIC) or minimum bactericidal concentration (MBC) determinations, which restricts quantitative comparison with existing literature.

All mechanical and biological assessments in the present study were conducted under in vitro conditions, without long-term cytotoxicity analysis or in vivo evaluation. Therefore, future work in our laboratory will focus on improving nanoparticle dispersion and undertaking extended biological investigations to better substantiate the potential clinical applications of the biosynthesized TiO₂ NPs. Additionally, variations in nanoparticle distribution within the GIC matrix may have influenced the reproducibility of the observed outcomes, and these observations will be critically addressed in our upcoming work.

## Conclusions

The current study showed that biosynthesized TiO₂ NPs can modestly enhance the performance of type-II GIC. At a loading of 100 mg/g, the NPs significantly reduced setting time, improved compressive strength, and produced a small but statistically significant reduction in *S. mutans* and *L. acidophilus* colony counts, with microwave-synthesized TiO₂ (F5) showing better results. Lower concentrations (50 mg/g) did not meaningfully alter mechanical or antibacterial behavior, and antioxidant activity remained low and statistically insignificant across all groups.

While these findings indicate potential benefits, they are limited to short-term in vitro conditions. Variability in nanoparticle dispersion and the absence of long-term cytotoxicity, MBC and MIC assays, and in vivo validation will be conducted in our further experiments to translate this potential material into real-time clinical applications.

## Appendices

Hydrothermal synthesis of titanium dioxide nanoparticles (TiO₂ NPs)

Preparation of Plant Extract

Fresh *Piper betle* leaves (500 g) were collected from Gudithota plantation, Tarikere, Chikkamagaluru District, Karnataka, India. The leaves were washed, shade-dried for 2-3 days, pulverized, sieved, and stored in an air-tight container. For extract preparation, 30 g of leaf powder was Soxhlet-extracted using 300 ml of ultrapure water, concentrated by rotary evaporation, and dried at 50°C for eight hours to obtain a green extract powder, which was stored at 4°C.

*Synthesis of TiO*₂* NPs*

For nanoparticle synthesis, TiCl₄ precursor was prepared by dissolving 7.99 g (1 M) of TiCl₄ in 100 ml of ultrapure water and stirring at 50°C for one hour. A freshly prepared 10% (w/v) *P. betle* aqueous extract (50 ml) was added dropwise to the precursor under continuous stirring. NaOH stock solution (1.5 g in 10 ml water) was added in 0.5 ml increments (total 3.5 ml) until a yellowish-white precipitate formed. The mixture was left undisturbed overnight, and the supernatant was decanted.

The precipitate was transferred to Teflon-lined stainless steel autoclaves and hydrothermally treated at 180°C for 18 hours. After cooling overnight, the brownish mixture containing a dark amber precipitate was washed repeatedly with ultrapure water and absolute ethanol and then dried at 100°C for 10 hours. The resulting amber-colored bioreduced TiO₂ NPs were ground using an agate mortar and stored at room temperature.

Results of Characterization 

X-ray diffraction (XRD) analysis confirmed the formation of pure anatase TiO₂ NPs with characteristic peaks matching JCPDS 21-1272 (Joint Committee on Powder Diffraction Standards) and an average crystallite size of ~23 nm. Fourier transform infrared spectroscopy (FTIR) spectra indicated the involvement of phytochemicals in NP formation, showing -OH, C-O, aldehyde, and C=O functional groups, along with a characteristic O-Ti-O band at 612 cm⁻¹. Ultraviolet-visible (UV-Vis) spectrophotometry exhibited absorption between 220 and 350 nm with a slight blue shift compared to commercial TiO₂. Scanning electron microscopy (SEM) images revealed agglomerated clusters of nearly spherical particles, and dynamic light scattering (DLS) showed a monomodal distribution of the particles with an average hydrodynamic diameter of ~39 nm. Energy-dispersive spectroscopy (EDS) confirmed the elemental composition consisting only of Ti and O, along with a trace amount of C, indicating the successful bioreduction and formation of phase-pure TiO₂ NPs with a coating of unreacted plant organics.

Microwave-assisted synthesis and characterization of TiO₂ NPs

Preparation of Ocimum sanctum Hydroalcoholic Extract

About 250 g of fresh leaves of *O. sanctum* were procured from the neighboring market, cleaned, washed, and dried in shade for 4-5 days. Dried leaves were pulverized using an electronic blender, sieved, and stored in an air-tight bottle. About 10 g of powder was dispensed inside a clean muslin cloth and tied. The hydro-alcohol solution was prepared by mixing 70 ml of ethyl alcohol with 30 ml of ultrapure water to make it 100 ml in total. The plant powder, which was packed inside a muslin cloth, was dropped into a 500 ml capacity reagent bottle. One hundred milliliters of hydro-alcoholic solution was poured into a bottle, and the lid was closed tightly. The bottle was covered with aluminum foil and kept in a dark place for five days with intermittent shaking. Further, the liquid was strained using cheesecloth, and the collected bottle green-colored liquid was dried at 45-50°C to extract the powder, which was ground and preserved in an air-tight container.

*Microwave-Assisted Synthesis of TiO*₂* NPs*

The titanium tetrachloride (99.9%) precursor material was obtained from Sigma Aldrich. 1 M of TiCl4 was dissolved in 100 ml of ice-cold ultrapure water. Once dissolved, the mixture was kept for continuous magnetic stirring at room temperature. Twenty milliliters of hydroalcoholic extract of *O. sanctum* was added dropwise at a pHof 6.7. The stirring process was continued until a light brown-colored precipitate settled at the bottom of the beaker. The supernatant was decanted, and the precipitate was transferred to a 100 ml capacity microwave-resistant reagent bottle. To this, 30 ml of ultrapure water was added, and the mixture was microwave-treated (LG-3288 domestic microwave) at 350 Watts. A total of seven cycles, each lasting for 60 seconds, was carried out. The color turned to dark brown, possibly due to the degradation of carbohydrates, glycosides, terpenoids, etc. Further, the ultrapure water in excess was added and allowed to cool overnight. The next day, the precipitate was washed several times with deionized water and dried at 200°C for 5-6 hours to obtain pale brown-colored TiO₂ NPs.

Results of Characterization 

XRD analysis showed broad but distinct anatase-phase peaks, with the prominent (101) reflection at 25.27°, confirming the formation of anatase TiO₂. FTIR spectra revealed O-H, C-N, and Ti-O-Ti/O-Ti-O bonding vibrations, indicating effective bioreduction and capping by *O. sanctum* phytochemicals. UV-Vis absorption showed a maximum near 375 nm, slightly blue-shifted relative to commercial TiO₂, suggesting reduced particle size. SEM analysis showed short, flat rod-like NPs with both dispersed and agglomerated regions, attributed to anisotropic growth directed by plant-derived chelators, and DLS confirmed a monomodal distribution with an average size of ~63 ± 9.2 nm in diameter and ~21 ± 3 nm in width.

Toxicological assessment of TiO₂ NPs synthesized by bio-assisted methods in comparison with commercially available TiO₂ NPs

The biocompatibility of the synthesized TiO₂ NPs was evaluated using hemolysis and cytotoxicity assays (using Balb/3T3 fibroblast cell lines). Hemolysis testing using chick blood indicated that both hydrothermally and microwave-assisted biosynthesized TiO₂ NPs exhibited significantly lower hemolytic activity compared to commercially procured TiO₂, with commercial TiO₂ causing ~4.4% hemolysis at 1 mg/ml, while the bio-assisted TiO₂ samples showed only minimal hemolysis (less than 3%) at the same concentration. Similarly, cytotoxicity assessment against Balb/3T3 fibroblast cell lines revealed that commercially available TiO₂ induced greater growth inhibition than the bio-assisted NPs. At 1 mg/ml, hydrothermally synthesized TiO₂ NPs caused ~30% growth inhibition, while microwave-assisted TiO₂ NPs caused ~28.6%, and lower nanoparticle concentrations supported increased fibroblast viability. Overall, the results demonstrate that bio-assisted synthesized TiO₂ NPs possess better hemocompatibility and lower cytotoxicity than commercially available TiO₂, indicating their suitability for biomedical and dental applications.

Discussion 

The biosynthesized TiO₂ NPs demonstrated favorable physicochemical and biological characteristics; however, several intrinsic limitations of plant-mediated synthesis must be recognized. Variability in the phytochemical profile of plant extracts influenced by environmental conditions, seasonal changes, plant maturity, and extraction parameters can significantly alter the concentration of reducing and stabilizing metabolites, resulting in batch-to-batch variations in particle size, morphology, and stability. Such variability may lead to deviations in particle size, morphology, or surface chemistry even under nominally identical conditions [31].

Additionally, despite the presence of phytoconstituents acting as natural stabilizers, limited control over particle clumping or secondary agglomeration remains a common drawback. The steric hindrance provided by plant metabolites is typically weak, failing to completely counterbalance attractive forces, resulting in aggregation during drying, purification, or storage [32,33].

Despite these limitations, the biological evaluation of the synthesized TiO₂ NPs demonstrated excellent biocompatibility compared to commercial TiO₂. Hemolysis studies using chick blood showed that commercial TiO₂ produced ~4.4% hemolysis at 1 mg/ml, whereas both hydrothermally and microwave-assisted biosynthesized TiO₂ NPs induced minimal hemolysis (<3%), indicating superior hemocompatibility. Cytotoxicity testing on Balb/3T3 fibroblast cells further supported this trend: commercial TiO₂ caused greater growth inhibition, while bio-assisted TiO₂ exhibited lower cytotoxicity, with hydrothermally synthesized NPs causing ~30% inhibition and microwave-assisted NPs ~28.6% at 1 mg/ml, with reduced effects at lower concentrations. These findings are consistent with previous reports that plant-mediated metal oxide NPs often retain phytochemical coatings that enhance cellular compatibility and reduce oxidative stress [34,35].

This supplementary section is adapted from the Ph.D. thesis titled "Bio-assisted Synthesis of Metal Oxide Nanomaterials and Their Applications in Dentistry", awarded to Dr. Shubha P by Mangalore University in 2023 (Notification No. MU/EXB/Ph.D./CR.92/Mat.Sc(239)/2017-18/E-25).
